# Hydroxychloroquine Patch Test Positivity in a Patient With Systemic Lupus Erythematosus

**DOI:** 10.1111/cod.70176

**Published:** 2026-04-29

**Authors:** Alexa G. Ries, Zachary Frost, Zachary Hopkins, Jamie P. Schlarbaum

**Affiliations:** ^1^ Spencer Fox Eccles School of Medicine University of Utah Salt Lake City Utah USA; ^2^ Noorda College of Osteopathic Medicine Provo Utah USA; ^3^ Department of Dermatology University of Utah Salt Lake City Utah USA

**Keywords:** case report, delayed hypersensitivity, hydroxychloroquine, patch test, systemic lupus erythematosus, type IV hypersensitivity

Hydroxychloroquine (HCQ) is critical for treating autoimmune diseases such as systemic lupus erythematosus (SLE). Hypersensitivity reactions, often in the setting of other initiated medications, may create diagnostic and therapeutic challenges. This case demonstrates the diagnostic value of patch testing for HCQ allergy in SLE.

## 1 Case Report

**FIGURE 1:**
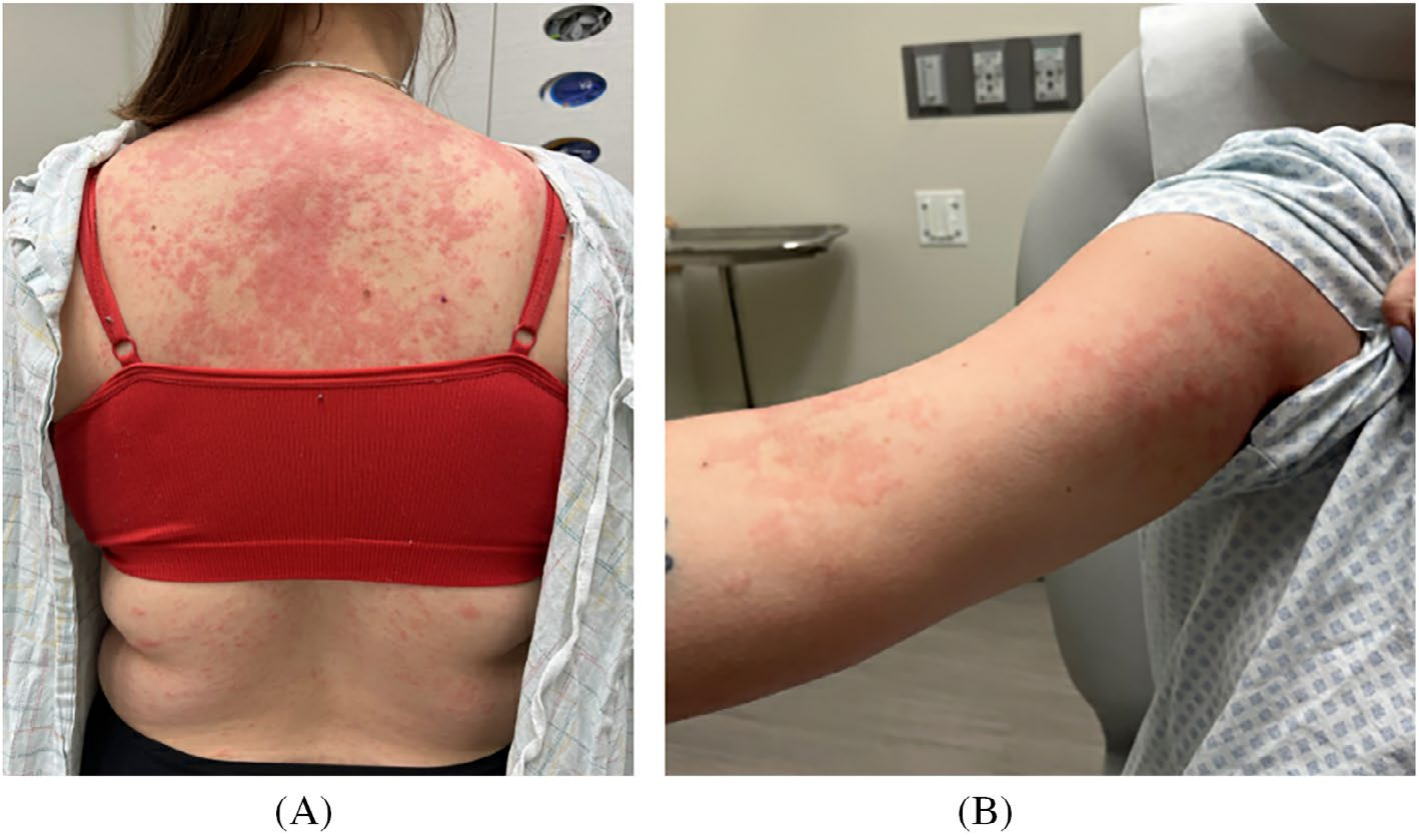
Red macules and papules coalescing into plaques on the (A) shoulders and back (B) right inner upper arm.

A 29‐year‐old woman with SLE developed a generalized rash 1 week after starting HCQ 200 mg and 3 weeks after starting meloxicam 15 mg daily. This pruritic morbilliform rash began on the soles before spreading to the extremities and trunk (Figure 1). She denied systemic symptoms. She reported previous cutaneous reactions to lamotrigine, amoxicillin and valproic acid.

Her complete blood count was within normal limits and without peripheral eosinophilia (37/mm^3^), alongside normal liver and renal function tests. Skin biopsies of the left upper back and left arm demonstrated spongiotic dermatitis with eosinophils and focal interface changes, most consistent with a drug eruption. The patient's European Registry of Severe Cutaneous Adverse Reactions (RegiSCAR) score was −1, suggesting a simple morbilliform drug eruption as the underlying diagnosis. Both meloxicam and HCQ were stopped. Symptomatic treatment with topical corticosteroids, calcineurin inhibitors and a prednisone taper was given and the rash cleared within 2–3 weeks.

**FIGURE 2:**
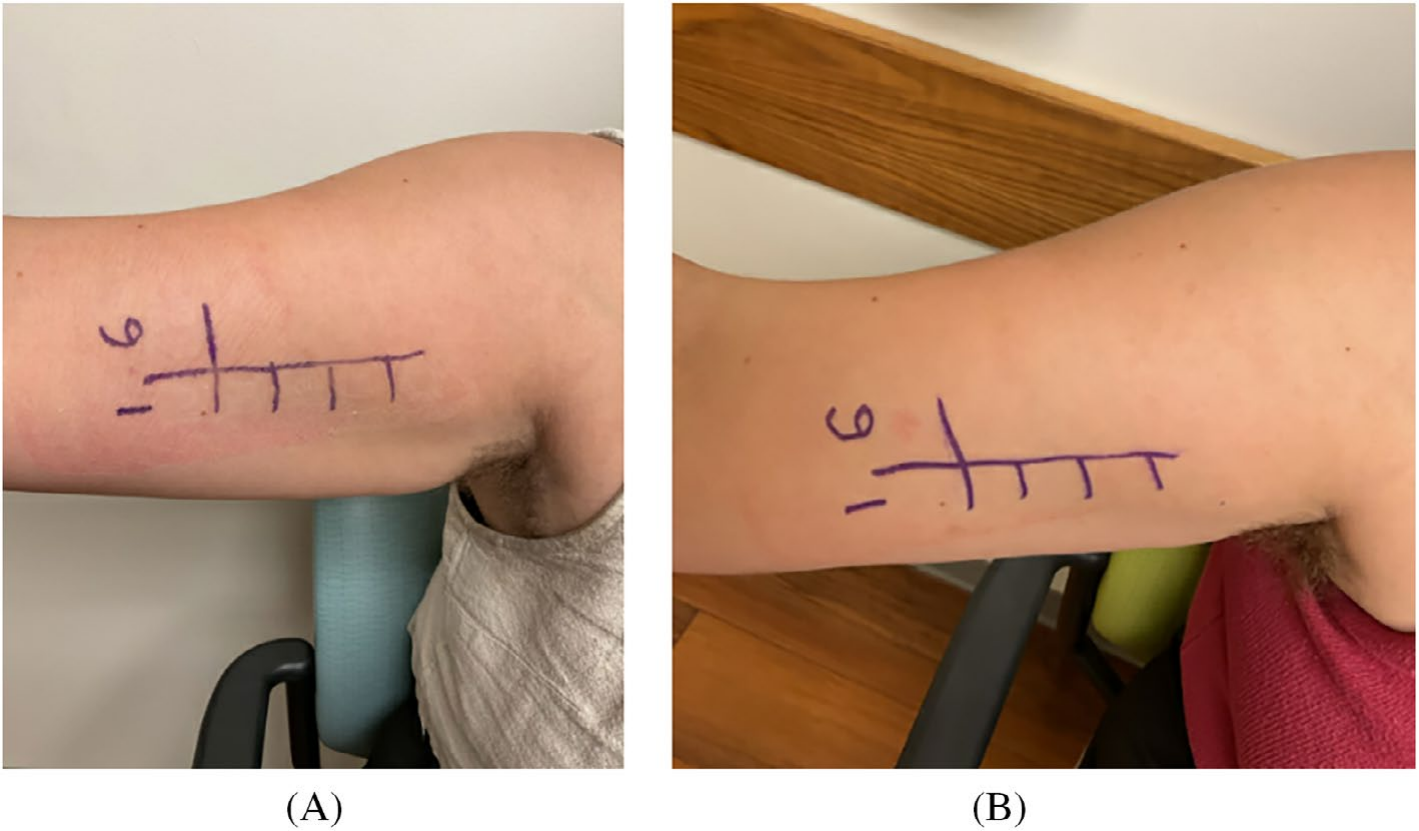
(A) Negative patch testing of right inner upper arm at 48 h read. (B) Mild (1+) positive patch testing for hydroxychloroquine (test position 6) and negative meloxicam (test position 3 and 4) at final read on Day 5.

Given the importance of HCQ for the patient's underlying SLE and her propensity for cutaneous adverse drug reactions, drug patch testing was performed 2 months after rash clearance. IQ chambers (Chemotechnique Diagnostics) and Hypafix tape (Leukoplast) were applied to the right upper arm (Figure 2), including clinic‐prepared HCQ 10% petrolatum (pet.), meloxicam (10% pet.), ibuprofen (10% pet., CAS 15687–27‐1, Chemotechnique Diagnostics) and ketoprofen (1% pet, CAS 22071–15‐4, Chemotechnique Diagnostics). Final read, per ICDRG standards, on Day 5 showed a mild 1+ reaction to HCQ. HCQ was thereby considered the causative agent of her previous eruption. A trial of an alternative antimalarial, chloroquine or quinacrine, was considered, though ultimately not performed as the patient was lost to follow‐up [1]. Sensitivity of meloxicam patch testing is not well‐defined; therefore, if needed, a 4–8 week oral NSAID monotherapy trial was recommended to ensure tolerability.

## 2 Discussion

About 10% of patients receiving HCQ develop hypersensitivity skin eruptions, most commonly a generalized morbilliform rash [2]. While positive drug patch testing has been reported, predominantly in acute generalized exanthematous pustulosis (AGEP) [2, 3, 4], patch testing has shown limited sensitivity in common delayed reactions [2]. A cohort (*N* = 11) of patients with HCQ hypersensitivity tolerated a trial of alternative antimalarials, chloroquine and quinacrine, suggesting a 4–8 week trial may be reasonable [1]; few antimalarial oral challenge and desensitization protocols are otherwise reported [5]. Therefore, patch testing to HCQ may be helpful in establishing next management steps [6, 7]. Drug patch testing is limited by low sensitivity and positivity rates are highly dependent on the medication and type of cutaneous adverse reaction [4, 7]; however, its occasional positivity, as demonstrated in our patient with SLE, underscores its potential value in clarifying the causative agent amidst polypharmacy as well as an opportunity to trial alternative medications.

## Author Contributions

**Alexa G. Ries:** conceptualization, writing – original draft, writing – review and editing, project administration. **Zachary Frost:** writing – original draft, writing – review and editing. **Zachary Hopkins:** conceptualization, investigation, writing – original draft, writing – review and editing, supervision. **Jamie P. Schlarbaum:** conceptualization, investigation, writing – original draft, writing – review and editing, supervision.

## Funding

The authors have nothing to report.

## Consent

We have received and archived written patient consent.

## Conflicts of Interest

Z.H. has received consulting fees from Priovant Therapeutics and is supported by a career development award from the Dermatology Foundation.

## Supporting information


**DATA S1:** Checklist for submission of case reports as ‘contact points’ [1].

## Data Availability

The data that support the findings of this study are available on request from the corresponding author. The data are not publicly available due to privacy or ethical restrictions.
